
JOURNAL INFORMATION
==============================
NLM Title Abbreviation: ScientificWorldJournal
Journal ID: ScientificWorldJournal
NLM Journal ID: 101131163
Journal ID: TSWJ

The Scientific World Journal

ISSN: 2356-6140
EISSN: 1537-744X
Publisher: Wiley

ARTICLE INFORMATION
==============================
PMCID: PMC5917318
PMID: 16830049
DOI: 10.1100/tsw.2006.166
Article ID: 947578
Article version: 1
Subjects: Directions in Science

Correlating Yeast Cell Stress Physiology to Changes in the Cell Surface Morphology: Atomic Force Microscopic Studies

Canetta Elisabetta 1
Walker Graeme M. 2
Adya Ashok K. 1 *
1 Condensed Matter Group and BIONTH (Bio- and Nano- Technologies for Health) Centre, School of Contemporary Sciences, University of Abertay Dundee, Bell Street, Dundee DD1 1HG, Scotland
2 Abertay Centre for Environment, School of Contemporary Sciences, University of Abertay Dundee, Bell Street, Dundee DD1 1HG, Scotland
*Ashok K. Adya: a.k.adya@abertay.ac.ukAcademic Editor: Robert P. Learmonth

Publication date: 2006
Electronic publication date: 2006 Jul 6
Volume: 6
First page: 777
Last page: 780
Received 2006 Apr 18; Revised 2006 Jun 28; Accepted 2006 Jun 29
Copyright: Copyright © 2006 Elisabetta Canetta et al.
Copyright year: 2006
License: This is an open access article distributed under the Creative Commons Attribution License, which permits unrestricted use, distribution, and reproduction in any medium, provided the original work is properly cited.
License URL: https://creativecommons.org/licenses/by/3.0/

Keywords: atomic force microscopy, AFM, microbiology, environmental stress, yeasts, morphology, Saccharomyces cerevisiae, Schizosaccharomyces pombe

Funding: University of Abertay Dundee

==============================
Directions in Science
*TheScientificWorldJOURNAL* (2006) 6, 777–780
ISSN 1537-744X; DOI 10.1100/tsw.2006.166

### Correlating Yeast Cell Stress Physiology to Changes in the Cell Surface Morphology: Atomic Force Microscopic Studies

Elisabetta Canetta[1], Graeme M. Walker[2], and Ashok K. Adya[1,]*
*[1]Condensed Matter Group and BIONTH (Bio- and Nano- Technologies for Health) Centre, [2]Abertay Centre for Environment, School of Contemporary Sciences, University of Abertay Dundee, Bell Street, Dundee DD1 1HG, Scotland*

**E-mail**: a.k.adya@abertay.ac.uk; g.walker@abertay.ac.uk; e.canetta@abertay.ac.uk

*Received April 18, 2006; Revised June 28, 2006; Accepted June 29, 2006; Published July 6, 2006*

**KEYWORDS:** atomic force microscopy, AFM, microbiology, environmental stress, yeasts, morphology, *Saccharomyces cerevisiae*, *Schizosaccharomyces pombe*

Yeasts are unicellular haploid fungi that help us to bake bread and ferment alcoholic beverages, but in some cases, they can cause infections that are sometimes fatal. Over 1000 different known species of yeasts are widely distributed in nature[1]. Although the majority of these (e.g., *Saccharomyces cerevisiae*) live in the general environment, a few species (e.g., *Candida*) are associated with humans and sometimes become pathogenic[1]. This occurs when our immune system weakens and *Candida* yeasts start to grow in an uncontrolled way, thereby causing candidiasis and nosocomial infections that result in high patient morbidity and mortality rates[2].

It is important to know more about yeasts, with the twofold aim to either exploit them better (brewing and baking industry) or to defend us more efficiently against them (medical research). In yeast biotechnology, the responses of yeasts to environmental stresses, such as physical (e.g., temperature and osmotic shock, desiccation/dehydration, high hydrostatic/gaseous pressures, gravity force/shear stress, and radiation), chemical (e.g., ethanol, nutrient limitation/starvation, oxidative and metal ion stress, pH shock, and chemical mutagenesis), and biological (e.g., cellular ageing, genotypic changes, and competition from other organisms), have been investigated[1,3,4]. A deeper characterisation of such responses, of the mechanisms involved in sensing a stress, and of the resulting changes in cell physiology and morphology is crucial for understanding how cells adapt and survive under adverse conditions. These studies are also important in order to understand the phenomenon of cellular ageing and cancer when using yeasts as model eukaryotes. However, for the above characterisation, researchers have so far mainly used biochemical assays to understand the yeasts' response to variety of stresses[5]. The same is true in medicine where microbiologists have extensively studied the metabolic activity of pathogenic yeasts (e.g., *Candida*) and their adhesive properties to medical devices by using only biochemical assays[6,7]. It is only recently that biophysical tools, such as atomic force microscopy (AFM) or optical tweezers, have started to be employed in yeast research[8]. In particular, the use of AFM has proved to be very powerful to study the biophysical (adhesive) properties of yeasts[9], their aggregation[10], and the nanomechanical dynamic motion of their cell walls[11].

Investigation of the adhesion of yeasts to a surface, a mammalian or bacterial cell, is crucial in order to solve the problems created in: (1) bioprocess engineering[9] (e.g., adhesion of yeasts to pipelines and heat exchangers), (2) medicine[7] (e.g., candidiasis and nosocomial infections), and (3) dentistry[12] (e.g., formation of biofilms on teeth due to bacteria adhesion or yeasts-bacteria coadhesion). The

*Corresponding author.
©2006 with author.
Published by TheScientificWorld, Ltd.; www.thescientificworld.com aggregation of yeast cells due to specific interactions plays a crucial role in the natural environment (e.g., bacteria/fungi adhesion to animal and plant tissues), medicine (e.g., formation of biofilms), and biotechnology (e.g., reversible aggregation of brewing yeasts during the fermentation process)[13]. A deeper knowledge of the specific receptor-ligand interactions involved in the phenomenon of yeast aggregation is needed to elucidate, for example, the flocculation of yeast cells. Many biological processes (e.g., passive diffusion of nutrients and transport of chemicals across the cell wall) that occur inside the yeast cells rely on the properties of the cell wall, thereby, necessitating the study of the dynamic nanomechanical activity of yeast cell wall.

Generally speaking, AFM[14] is a powerful tool for 3-D visualisation of a biological sample under physiological conditions at high resolution, and for measurements of their biophysical and biomechanical (elastic and viscoelastic) properties without destroying the specimens[15]. Over the past few years, the use of AFM to investigate the properties of microbial surfaces (e.g., bacteria and yeast cells) at nano-scale has largely increased.

Ahimou et al.[16] analysed the surface topography of living *S. cerevisiae* cells, and monitored enzyme digestion of the cell wall in real-time by immobilising yeasts in porous membranes and visualising them under physiological conditions. Roughness analysis of the AFM images showed the surfaces of yeasts to be quite smooth. Bowen et al.[9] used AFM to measure the adhesive force of metabolically active *S. cerevisiae* yeasts onto hydrophilic, hydrophobic, and protein-coated mica surfaces in an aqueous environment. They immobilised individual yeasts at the end of AFM tipless cantilevers and allowed them to come into contact with the mica surfaces. The maximum value for adhesion was obtained when the yeasts were allowed to adhere onto the hydrophobic mica surface. Also, the cell age was found to play an important role in yeast-surface adhesion. Additionally, yeast cells in stationary phase were found to adhere most strongly to the mica surface. Touhami et al.[10] measured the individual lectin-carbohydrate interactions, involved in the phenomenon of reversible aggregation (flocculation) of *S. carlsbergensis* yeasts, by functionalising an AFM cantilever with carbohydrates and allowing it to approach and come in contact with the surface of an individual yeast cell on which the active lectins were expressed. AFM force spectroscopy experiments performed in flocculating conditions of these yeasts showed the occurrence of specific interactions between individual cell-surface lectins and glucose residues. Pelling et al.[11] carried out AFM experiments to study the local nanomechanical motion of the living *S. cerevisiae* cell walls. Yeasts were immobilised in a polycarbonate membrane to work under physiological conditions. The cell wall of *S. cerevisiae* was found to exhibit local nanomechanical motion at a temperature-dependent characteristic frequency, which increased with the temperature. The mechanical oscillation of the *S. cerevisiae* cell wall was linked to the active metabolic processes taking place inside the cell.

Our group at the BIONTH Centre (UAD) is currently using the AFM technique to study the morphological and biophysical properties of a variety of microbial systems ranging from yeast cells to virus-encoded proteins[17]. In two recent studies published in *FEMS Yeast Research*[18] and *FEMS Microbiology Letters*[19], we investigated by AFM topographic analyses the morphological changes of one strain each of *S. cerevisiae* (NCYC 1681) and *Schizosaccharomyces pombe* (DVPB 1354) in response to the imposed thermal, osmotic, and ethanol stresses. The fine changes occurring on the cell walls in stressed yeasts were analysed by using AFM in conjunction with measurements of cell viability and mean cell volume. The two types of yeasts were exposed to 0, 40, 50, and 90°C (thermostress), 10, 20, 30, and 40% w/v sorbitol (osmostress), and 10, 20, and 30% v/v ethanol (ethanol shock) concentrations for different times of up to 1 h. Unstressed yeasts incubated at room temperature (25°C) acted as controls for the thermal, osmotic, and ethanol shocks.

Unstressed and stressed yeasts were fixed onto a glass slide by allowing them to gently dry overnight at room temperature. Although it is now possible to perform AFM experiments on yeasts in their native hydrated state, one is faced with the additional problem of immobilising the cells on solid substrates in liquid environment. Different methods, such as using porous membranes of different porosity[8], are being tried (see above) to confine the individual cells in the pores. We believe that entrapping cells inside confined geometries will expose them to additional stress in confinement[20], which even in pure liquids is known to change their structure substantially. Nevertheless, since we analysed and interpreted our results of changes in the surface morphology of the stressed cells relative to the unstressed cells, which were allowed to dry gently in air in the same way as the stressed cells, the relative changes were truly due to the additional stress imposed and not due to the air-drying method we used to fix the cells. It has also been shown recently[21] that imaging in air of microbial cell surfaces reveal many morphological details that remain missing in the cells imaged in liquid. Pelling et al.[11] proved that the morphology of the yeast surface does not really change when dried gently in air. Thus, fixation of yeasts by trapping them in porous membranes appears to be essential only when the biophysical and biomechanical properties of yeast cells are to be measured because for such studies, the cells must be kept alive in order to maintain their metabolic activity.

For both the *S. cerevisiae* and *Schiz. pombe* strains, cell viability was found to decrease quickly with increase in the exposure time, the temperature (for thermal stress), the sorbitol concentration (for osmotic stress), and the ethanol concentration (for ethanol stress)[18,19]. The observed effects of both thermal and osmotic stresses on cell physiology (cell viability and mean cell volume) and morphology (surface roughness and bearing volume analyses of the AFM images) indicated that *Schiz. pombe* (strain DVPB 1354) was probably more resistant to physical stresses than *S. cerevisiae* (strain NCYC 1681). Conversely, the effects of ethanol toxicity were much more detrimental to *Schiz. pombe* than *S. cerevisiae*. These results, in particular for *S. cerevisiae*, may be of interest in yeast biotechnology to further improve the fermentation and winemaking processes.

It is clear from the above discussion that AFM has become a very powerful tool in a relatively short time to study yeasts. AFM imaging has been used to visualise the conformational changes of yeast cell surfaces[18,19], while AFM force spectroscopy has allowed investigating the biophysical and nanomechanical properties of yeast cells[11]. In addition, the very high resolution of AFM has allowed real-time investigation of the enzyme digestion of the yeast cell wall[16]. The capability of AFM to measure interaction forces at a single molecule level has opened new horizons in the study of aggregation of yeasts[10].

A recent application of AFM that should be very interesting in yeast research is the nanomanipulation of biological systems[22]. The possibility to combine AFM with other microscopic techniques, such as inverted (AFM-IM), fluorescence (AFM-FM)[23], and total internal reflection fluorescence (AFM-TIRFM)[24] microscopes, has added further dimensions to this already fast-growing field. In particular, AFM-FM and AFM-TIRFM systems increase the versatility of AFM for future applications to nanobiotechnology and yeast research by precise manipulation of single molecules. Also, nanosurgery of living cells is a new field that will catch up very fast. The development of advanced techniques to modify the shapes of AFM tips to obtain ultrathin needles that can be used to penetrate at precise positions inside the cells will allow us to modify the cell's functions[25]. This will have a huge impact in nanomedicine. In yeast research, the nanosurgery technique can be used, for example, to inject antifungal drugs in *Candida* yeasts in order to study the changes in their morphological, biophysical, and biomechanical properties. These studies will improve our knowledge of the complex processes, underlying the development/spread of *Candida* infections that will ultimately help to find efficacious drugs to cure/prevent them. Another implementation of the AFM technique will be the combination of AFM with optical tweezers (AFM-OT) that should permit us, in future, to increase the range of detectable forces to ~ 0 pN to 0.1 μN. It would also be possible to trap a single molecule or a living cell with OT and visualise with AFM, in real-time, the changes in the sample morphology while it undergoes mechanical stresses, such as stretching or compression.

These are just some examples of new developments in the AFM technology. Our recent results using the AFM have helped us to correlate the yeast cell stress physiology to the yeast cell surface morphology. It is clear that the applications of AFM and other above-mentioned techniques, either in isolation or in conjunction, to biological systems can reveal their insightful details.

### ACKNOWLEDGEMENTS

We wish to acknowledge the support from the University of Abertay Dundee (UAD) for this work. EC is grateful to the UAD for the award of a postdoctoral research assistantship (PDRA).

### REFERENCES

1. Walker, G.M. (1998) *Yeast Physiology and Biotechnology*. John Wiley & Sons, Chichester, West Sussex, U.K.
2. Hobson, R.P. (2003) The global epidemiology of invasive *Candida* infections-is the tide turning? *J. Hosp. Infect.* **55**, 159–168.
3. Piper, P.W. (1995) The heat shock and ethanol stress responses of yeast exhibit extensive similarity and functional overlap. *FEMS Microbiol. Lett.* **134**, 121–127.
4. Cabral, M.G., Viegas, C.A., and Sá-Correia, I. (2001) Mechanisms underlying the acquisition of resistance to octanoic-acid-induced-death following exposure of *Saccharomyces cerevisiae* to mild stress imposed by octanoic acid or ethanol. *Arch. Microbiol.* **175**, 301–307.
5. Estruch, F. (2000) Stress-controlled transcription factors, stress-induced genes and stress tolerance in budding yeasts. *FEMS Microbiol. Rev.* **24**, 469–486.
6. Chandra, J., Kuhn, D.M., Mukherjee, P.K., Hoyer, L.L., McCormick, T., and Ghannoum, M.A. (2001) Biofilm formation by the fungal pathogen *Candida albicans*: development, architecture, and drug resistance. *J. Bacteriol.* **183**, 5385–5394.
7. Brun, S., Dalle, F., Saulnier, P., Renier, G., Bonnin, A., Chabasse, D., and Bouchara, J.P. (2005) Biological consequences of petite mutations in *Candida glabrata*. *J. Antimicrob. Chemother.* **56**, 307–314.
8. Dufrêne, Y.F. (2002) Atomic force microscopy, a powerful tool in microbiology. *J. Bacteriol.* **184**, 5205–5213.
9. Bowen, W.R., Lovitt, R.W., and Wright, C.J. (2001) Atomic force microscopy study of the adhesion of *Saccharomyces cerevisiae*. *J. Colloid Interf. Sci.* **237**, 54–61.
10. Touhami, A., Hoffman, B., Vasella, A., Denis, F.A., and Dufrêne, Y.F. (2003) Aggregation of yeast cells: direct measurement of discrete lectin-carbohydrate interactions. *Microbiology* **149**, 2873–2878.
11. Pelling, A.E., Sehati, S., Gralla, E.B., Valentine, J.S., and Gimzewski, J.K. (2004) Local nanomechanical motion of the cell wall of *Saccharomyces cerevisiae*. *Science* **305**, 1147–1150.
12. Whittaker, C.J., Klier, C.M., and Kolenbrander, P.E. (1996) Mechanism of adhesion by oral bacteria. *Annu. Rev. Microbiol.* **50**, 513–552.
13. Watnick, P. and Kolter, R. (2000) Biofilm, city of microbes. *J. Bacteriol.* **182**, 2675–2679.
14. Binning, G., Quate, C.F., and Gerber, C. (1986) Atomic force microscope. *Phys. Rev. Lett.* **56**, 930–933.
15. Canetta, E. and Adya, A.K. (2005) Atomic force microscopy: applications to nanobiotechnology. *J. Indian Chem. Soc.* **82**, 1147–1172.
16. Ahimou, F., Touhami, A., and Dufrêne, Y.F. (2003) Real-time imaging of the surface topography of living yeast cells by atomic force microscopy. *Yeast* **20**, 25–30.
17. Canetta, E. and Adya, A.K. (2006) Morphological and biophysical properties of cells and proteins by atomic force microscopy. In Proceedings of VIII Annual Linz Winter Workshop on Advances in Single-Molecule Research for Biology and Nanoscience, in press.
18. Adya, A.K., Canetta, E., and Walker, G.M. (2006) Atomic force microscopic study of the influence of physical stresses on *Saccharomyces cerevisiae* and *Schizosaccharomyces pombe*. *FEMS Yeast Res.* **6**, 120–128.
19. Canetta, E., Adya, A.K., and Walker, G.M. (2006) Atomic force microscopic study of the effects of ethanol on yeast cell surface morphology. *FEMS Microbiol. Lett.* **255**, 308–315.
20. Morineau, D., Xia, Y.D., and Alba-Simionesco, C. (2002) Finite-size and surface effects on the glass transition of liquid toluene confined in cylindrical mesopores. *J. Chem. Phys.* **117**, 8966–8972.
21. Doktycz, M.J., Sullivan, C.J., Hoyt, P.R., Pelletier, D.A., and Allison, D.P. (2003) AFM imaging of bacteria in liquid media immobilised on gelatin coated mica surfaces. *Ultramicroscopy* **97**, 209–216.
22. Fotiadis, D., Scheuring, S., Müller, S., Engel, A., and Müller, D. (2005) Imaging and nanomanipulation of biological structures with the AFM. *Micron* **33**, 385–397.
23. Hards, A., Zhou, C., Seitz, M., Bräuchle, C., and Zumbusch, A. (2005) Simultaneous AFM manipulation and fluorescence imaging of single DNA strands. *Chem. Phys. Chem.* **6**, 534–540.
24. Nishida, S., Funabashi, Y., and Ikai, A. (2002) Combination of AFM with an objective-type total internal reflection fluorescence microscope (TIRFM) for nanomanipulation of single cells. *Ultramicroscopy* **91**, 269–274.
25. Obataya, I., Nakamura, C., Han, S., Nakamura, N., and Miyake, Y. (2005) Nanoscale operation of a living cell using an atomic force microscope with a nanoneedle. *Nano Lett.* **25**, 27–30.

**This article should be cited as follows:**

Canetta, E., Walker, G.M., and Adya, A.K. (2006) Correlating yeast cell stress physiology to changes in the cell surface morphology: atomic force microscopic studies**.** *TheScientificWorldJOURNAL* **6,** 777–780. DOI 10.1100/tsw.2006.166.
